# Neutrophils in HNSCC Can Be Associated with Both a Worse or Favorable Prognosis

**DOI:** 10.3390/biom14020205

**Published:** 2024-02-09

**Authors:** Hendrik Brunkhorst, Sören Schnellhardt, Maike Büttner-Herold, Christoph Daniel, Rainer Fietkau, Luitpold V. Distel

**Affiliations:** 1 Department of Radiation Oncology, Universitätsklinikum Erlangen, Friedrich-Alexander-Universität Erlangen-Nürnberg, 91054 Erlangen, Germany; 2 Comprehensive Cancer Center Erlangen-EMN (CCC ER-EMN), 91054 Erlangen, Germany; 3 Department of Radiotherapy and Radiation Oncology, Saarland University Medical Center, 66421 Homburg, Germany; 4 Department of Nephropathology, Institute of Pathology, Universitätsklinikum Erlangen, Friedrich-Alexander-Universität Erlangen-Nürnberg, 91054 Erlangen, Germany

**Keywords:** CD66b+, neutrophils, tumor-associated neutrophils, prognosis, HNSCC, head and neck cancer, hypopharynx, oropharynx, larynx, oral cavity

## Abstract

The prognostic significance of tumor-infiltrating neutrophils in head and neck squamous cell carcinoma (HNSCC) is poorly understood. It is unclear how the presence of neutrophils affects prognosis due to their polarization into cytotoxic N1 or immunosuppressive N2. Therefore, we determined the number of CD66b+ neutrophil granulocytes separately in the stromal and epithelial compartments in cancer tissues from 397 patients with HNSCC. Tumor samples from six historical patient groups were processed into tissue microarrays and stained immunohistochemically. In total, 21.9% were HPV positive (p16+). Neutrophil counts were much lower in the stromal compartment (372 ± 812) than in the epithelial cancer compartment (1040 ± 1477) (*p* < 0.001), with large differences between groups. In three groups with high neutrophil infiltration, high rates were associated with a favorable prognosis, whereas in two groups, high rates were a negative prognostic factor. In p16- oropharyngeal and hypopharyngeal cancer high infiltration was associated with a favorable prognosis. Cancers with an exclusion of neutrophils in the epithelial compartment were associated with improved prognosis. In oropharyngeal and hypopharyngeal HPV-negative cancer high neutrophil infiltration rates were clearly associated with prolonged survival. Neutrophil granulocytes in HNSCC may contribute to a favorable or unfavorable prognosis.

## 1. Introduction

Cancers of the oral cavity, oropharynx, hypopharynx, and larynx are a diverse group of malignancies with different etiologies and very different prognoses, despite their collective classification as head and neck squamous cell carcinoma (HNSCC). The major risk factors for HNSCC are tobacco use, excessive alcohol consumption, and persistent and recurrent infection with high-risk human papillomavirus (HPV) [1]. Air pollution, aging, poor oral hygiene, and a diet low in vegetables are minor risk factors [2]. In general, HNSCC tumors are highly infiltrated by immune cells. However, the extent and composition of the immune infiltrate varies by anatomic subsite and by etiology and etiologic agent [3,4,5]. The tumor microenvironment of HNSCC is highly immunosuppressive due to a wide spectrum of suppressive inflammatory cells [6,7]. Among these regulatory T cells, myeloid-derived suppressor cells and M2 macrophages may be the most common [8]. However, there are also immunologically “cold” tumors in HNSCC with only minor immune cell infiltration in both the stromal and epithelial compartments (“immune desert”) [9]. This demonstrates that infiltrating immune cells can contribute to both an anti-tumorigenic and a pro-tumorigenic environment. Additionally, there are tumors that are able to prevent immune cells from infiltrating into tumor epithelium (“immune excluded”), as well as “inflamed” tumors that are interspersed with immune cells [9]. Different mechanisms of immunosuppression are assumed in each immune phenotype and an improved understanding of these mechanisms is essential in the development of new therapeutic strategies [10].

The most common immune cell types in solid cancers are neutrophils [11]. However, data on the role of neutrophils in HNSCC are limited, as peripheral blood neutrophils have mainly been studied in the context of tumors and, to a much lesser extent, neutrophils within tumors [12]. Developing tumor cells that secrete factors such as IL-8, CCL4, or CCL5 may attract neutrophils or their precursors [13]. Thus, neutrophils infiltrate the tumor microenvironment. Under the action of mainly IFN-β, N1-polarized neutrophils are stimulated and N2-polarized neutrophils are inhibited [14]. TGF-β, on the other hand, inhibits N1 and promotes N2-polarization [15]. In this manner, neutrophils can be polarized into antitumor N1 neutrophils or protumor N2 neutrophils [15,16,17]. Tumor-associated neutrophils can regulate innate and adaptive immunity and play an important role in mediating T cell-independent antibody responses, as well as antigen presentation and T cell activation [18,19]. The concept of neutrophil polarization into N1 and N2 phenotypes is supported by several studies [20]. However, it is important to note that there are currently no validated surface markers for phenotypic identification of N1 and N2 neutrophils in histologic tissues [21].

As a consequence, neutrophils can reshape the tumor microenvironment as N1 by acting pro-inflammatory with anti-tumor properties [15,19], while N2 support tumor growth and angiogenesis and enhance the immunosuppressive tumor microenvironment [19,22]. Although N1 neutrophils are highly cytotoxic cells that show a mature phenotype and high immune-stimulating activity, they are only short living. In contrast, N2 neutrophils have a longer life span in which they can perform their immunosuppressive activity.

In patients with advanced oro- or hypopharyngeal HNSCC increased intratumoral infiltration with neutrophils was associated with reduced overall survival [23,24]. Therefore, we were interested in neutrophil infiltration and its prognostic significance in all anatomic subsites of advanced HNSCC.

## 2. Materials and Methods

Tumor tissue from 397 patients with partially locally advanced and/or metastatic head and neck squamous cell carcinoma (HNSCC) was the basis of our analysis (Table 1). The majority of all patients in our cohorts suffered from T3/4 cancers (61%) and UICC stage IV disease (45%), and most tumors were located in the oropharynx (51%). The total cohort consists of six groups which were established between 2007 and 2017 and included patients treated between 1998 and 2015. Four groups received adjuvant, one definitive and one neoadjuvant radiochemotherapy (Table 2). The median follow-up was 4.3 years. The use of formalin-fixed, paraffin-embedded material from the archives of the Institute of Pathology was approved by the Ethics Committee of the Friedrich-Alexander University of Erlangen–Nuremberg on 24 January 2005. Thus, no consent was required for the use of existing archived material. All patients gave informed consent to have their tissue and clinical data collected.

We used formalin-fixed, treatment-naive, paraffin-embedded biopsies or tumor resection specimens from 397 patients. The samples were processed into tissue microarrays (TMAs) with a diameter of 2 mm and stained with a mouse monoclonal anti-human CD66b antibody (clone G10F5, dilution 1:200) (555723, BD Biosciences, Heidelberg, Germany). CD66b was chosen because of its high affinity and specificity for cancer neutrophils [25]. Antibody labeling was performed with biotinylated goat anti-mouse IgM secondary antibody (Vector Laboratories, Newark, CA, USA) after removal of unbound antibody by washing with 50 mM Tris(hydroxymethyl)aminomethane buffer pH 7.6 supplemented with 0.1% Tween 20. Sections were processed with VectaStain ABC kit according to the manufacturer’s instructions using DABImmpact (both Vector Laboratories) as substrate. Finally, the sections were counterstained with hemalaun. They were dehydrated and coverslipped with Entellan (Merck KGaA, Darmstadt, Germany). CD66b+ cells were interpreted as neutrophils. Isotype controls were performed and showed no relevant staining (Supplementary Figure S1). p16 was routinely studied when the biopsy or tumor specimen was examined, and missing diagnoses were redetermined on the TMAs (Clone MIB-1, Agilent, Santa Clara, CA, USA).

Stained samples were scanned using a whole slide scanner (Axio imager Z2, Carl Zeiss GmbH, Oberkochen, Germany) at 400× magnification and analyzed using the image analysis software COUNT (BIOMAS Software, version 3.3; MSAB, Erlangen, Germany). Stromal and intraepithelial compartments were analyzed separately and the respective cell densities were calculated semi-automatically using BIOMAS. Neutrophils were scored as positive only if they met the following requirements: positive staining (brown) and positive (blue) staining of the nucleus, a size of at least 10 µm in diameter and an appropriate shape.

Overall survival, recurrence-free survival and metastasis-free survival were measured in survival analysis. Statistical analyses were performed using SPSS version 26 (IBM Inc., Chicago, IL, USA). The Kaplan–Meier method was used to generate survival plots. X-tile software Version 3.6.1 (Yale School of Medicine, New Haven, CT, USA) was used to determine optimal cut-off values for each cohort. An optimal cut-off point was calculated for each Kaplan–Meier plot. We used the log-rank test for the comparison of survival distributions, the Student’s *t*-test for comparison of means, and the chi-squared test for comparison of categorized items. *p* values < 0.05 were considered statistically significant. Hazard ratios of cell densities and clinicopathologic characteristics were calculated using the Cox proportional hazards model. The five covariates with the lowest univariate *p*-values were included in multivariate analyses. The assumption of the proportional hazards model was verified by visual inspection of the log-minus-log curves.

## 3. Results

### 3.1. Clinical Characterization of the Head and Neck Squamous Cell Carcinoma Cohort

**Figure 1:**
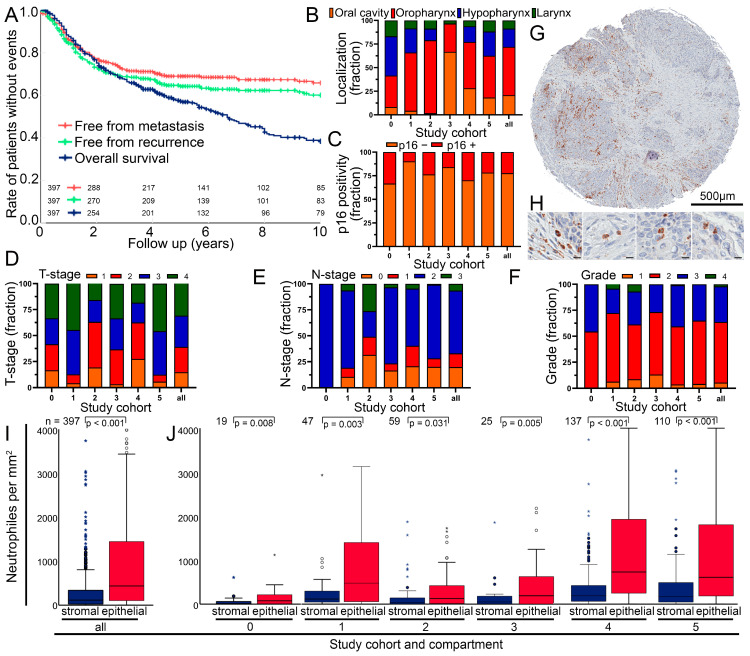
(**A**) Kaplan–Meier plots of 10-year survival of the analyzed cohort divided into overall survival, recurrence-free survival and metastasis-free survival. (**B**) Tumor localization, (**C**) p16 status, (**D**) T stage, (**E**) N stage and (**F**) tumor grade fraction in the subcohorts. (**G**) Representative image of a tissue spot from a tissue microarray (TMA) with distinct neutrophil granulocyte infiltration. (**H**) Close-up of neutrophil granulocytes from image (**G**). Scale bar indicates 10 µm (**I**) Boxplot of neutrophil density in stroma and epithelium. (**J**) Boxplots of neutrophil density in the subcohorts. Symbols indicate outliers.

A cohort of 397 patients suffering from head and neck squamous cell carcinoma was used to study the prognostic significance of tumor-infiltrating neutrophils. The median follow-up time was 4.3 years. There was a relatively even distribution of all T stages, the vast majority of patients (79.1%) had lymph node involvement and 1.3% suffered from distant metastasis (Table 1). The cohort consisted of patients from six different clinical studies conducted between 1984 and 2015 (Table 2). Patients in four trials were treated with surgery followed by radiotherapy or radiochemotherapy (RCT) (adjuvant), and patients in one trial were treated with RCT followed by surgery (neoadjuvant). In one trial, patients were treated with RCT alone (definitive). After ten years of follow-up, 60.1% of patients were free from recurrence and 65.9% were free from metastases. Overall survival at 10 years was 38.4% (Figure 1A).

### 3.2. Composition of the Cohort and Cell Numbers in the Different Tumor Sides

The location of the cancers in the oral cavity, oropharynx, hypopharynx and larynx varied greatly between the six cohorts (Table 1, Figure 1B). On average, 21.9% of cancers were p16 HPV positive (Figure 1C). T stage, N stage and grade varied widely between cohorts (Figure 1D–F). Age had no prognostic significance (*p* = 0.903), p16+ was prognostically very favorable (*p* = 0.001), and there was no difference between sex (*p* = 0.348) and localization (*p* = 0.343). The high T stage was clearly prognostically unfavorable (*p* = 0.030) and the N stage was only borderline significant (*p* = 0.127) (Supplementary Figure S2). Neutrophil granulocytes were stained by anti-CD66b antibodies (Figure 1G,H) and were counted semiautomatically separately in the stromal and intraepithelial compartments (Supplementary Figures S3 and S4). Cell densities in the stromal compartment were much lower (mean 372 neutrophils/mm^2^, SD ± 812) compared to the epithelial cancer compartment (mean 1040 neutrophils/mm^2^ ± 1477) (*p* < 0.001) (Figure 1I). This is unusual, as there are often more inflammatory cells in the stromal compartment. In these tissue sections, only 16.1% of samples had more neutrophils in the stromal than in the epithelial compartment. In all six studies, stromal counts were markedly lower than epithelial counts (*p* < 0.031) (Figure 1J). Of the six different cohorts, three were similar in terms of neutrophil infiltration. Study cohorts 0, 2 and 3 did not differ in the number of stromal neutrophils (*p* > 0.229) or epithelial neutrophils (*p* > 0.083). Compared to study cohorts 0, 2 and 3, neutrophil counts in cohorts 1, 4 and 5 were significantly higher (*p* < 0.001), but did not differ between the stromal (*p* > 0.466) or epithelial (*p* > 0.083) compartments.

### 3.3. Prognosis in the Different Cohorts According to Neutrophil Density in the Stromal and the Epithelial Compartments

**Figure 2:**
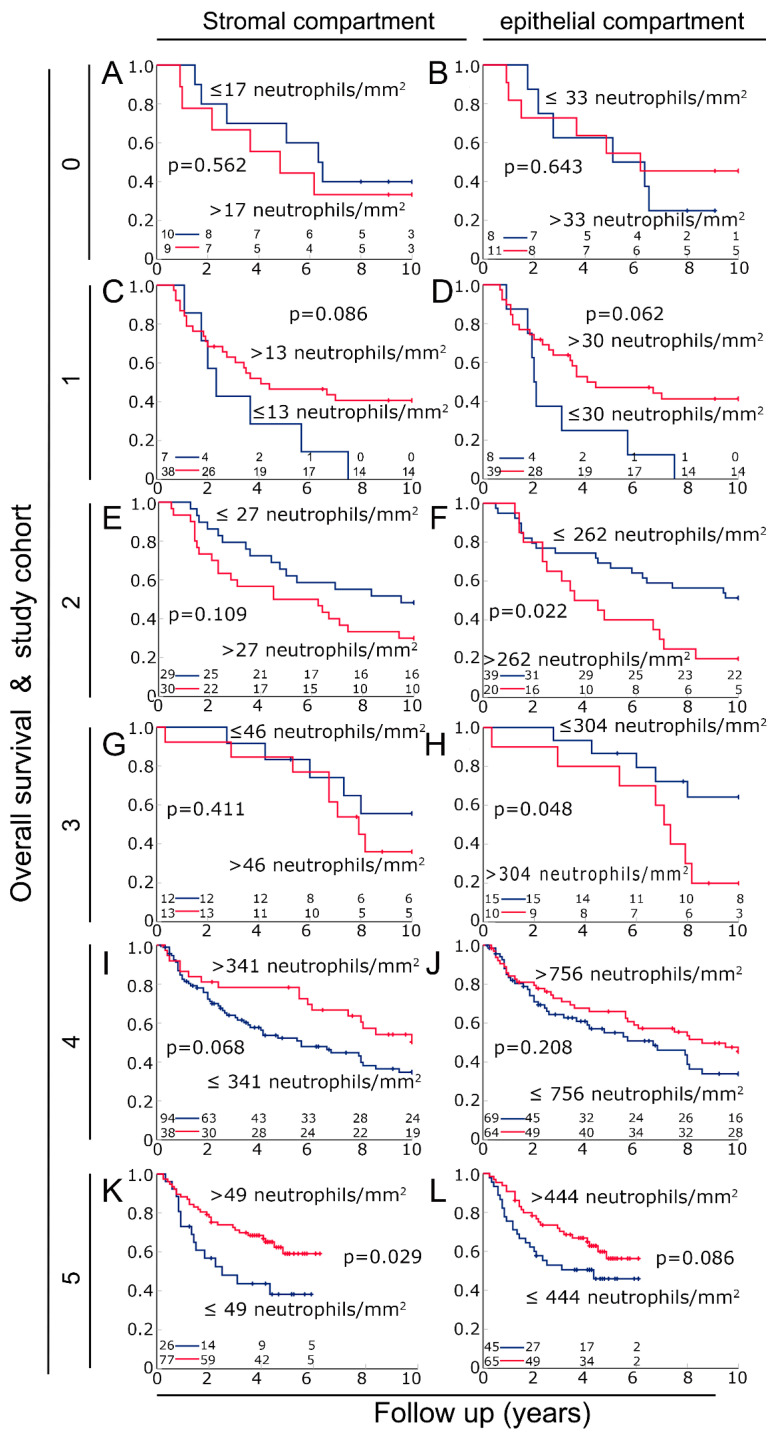
Overall survival of patients with HNSCC in six cohorts according to neutrophil densities in the stromal and epithelial compartments of the tumor using Kaplan–Meier plots. Cohort 0 (**A**) in the stromal and (**B**) in the epithelial compartments of the tumor. Cohort 1 (**C**) stromal and (**D**) epithelial compartments. Cohort 2 (**E**) stromal and (**F**) epithelial compartments. Cohort 3 (**G**) stromal and (**H**) epithelial compartments. Cohort 4 (**I**) stromal and (**J**) epithelial compartments. Cohort 5 (**K**) stromal and (**L**) epithelial compartments.

Next, we were interested in the prognostic significance of neutrophil granulocytes in the two compartments. Therefore, Kaplan–Meier plots were used to analyze overall survival (Figure 2). In cohort 0, neutrophil density had no prognostic significance in either the stromal (*p* = 0.562) or epithelial compartments (*p* = 0.643) (Figure 2A,B). Cohort 0 was therefore excluded from further analysis. In cohorts 1, 4 and 5, high numbers of tumor-infiltrating neutrophils had a non-significant association with favorable survival in both the stromal (*p* < 0.086) and epithelial compartments (*p* = 0.208) (Figure 2C,D,I–L). Conversely, in study cohorts 2 and 3, high neutrophil counts were associated with an unfavorable prognosis (Figure 2E–H). This association was stronger for the epithelial compartment (*p* < 0.048) than for the stromal compartment (*p* < 0.411).

### 3.4. Combining Cohorts into Groups with Favorable and Unfavorable Prognostic Significance of Neutrophils

**Figure 3:**
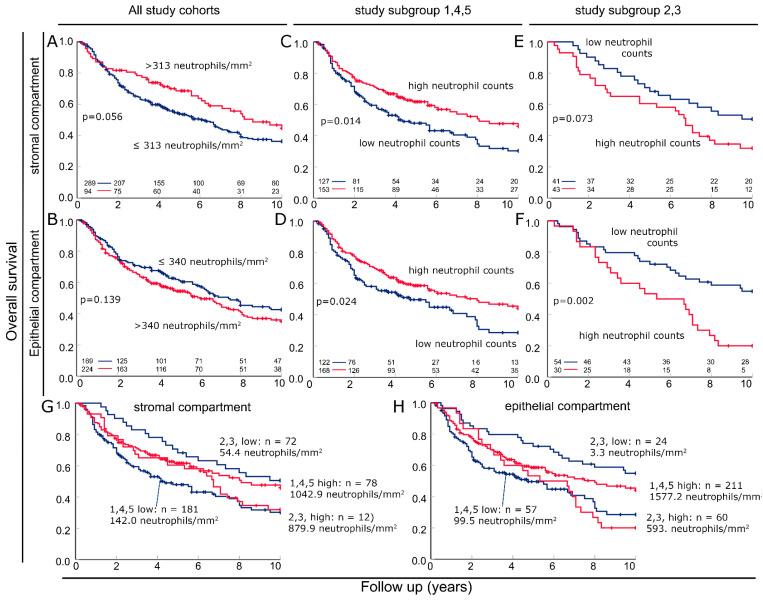
Subcohorts pooled in Kaplan–Meier curves according to favorable and unfavorable prognosis with high neutrophil infiltration in the stromal and epithelial compartments. Kaplan–Meier plots containing all subcohorts of overall survival according to neutrophil densities in (**A**) the stromal and (**B**) epithelial compartments. Kaplan–Meier plots containing cohorts 1, 4 and 5 of overall survival according to neutrophil densities in (**C**) stromal and (**D**) epithelial compartments. Kaplan–Meier plots containing subcohorts 2 and 3 of overall survival according to neutrophil densities in (**E**) stromal and (**F**) epithelial compartments. (**G**) Kaplan–Meier plot containing (**C**,**D**) of overall survival according to stromal neutrophil densities. (**H**) Kaplan–Meier plots containing (**E**,**F**) of overall survival according to epithelial neutrophil densities.

Combining all six cohorts, there was a slight association of high neutrophil density with favorable outcomes in the stromal compartment (*p* = 0.056) and a slight association with poor outcomes in the epithelial compartment (*p* = 0.139) (Figure 3A,B). The three cohorts 1, 4 and 5 were then combined into group 1,4,5, which had high neutrophil infiltration and a favorable prognosis associated with high neutrophil counts. There was a clear association between increased tumor-infiltrating neutrophils and prolonged survival in both the stromal compartment (*p* = 0.014) and the epithelial compartment (*p* = 0.024) (Figure 3C,D). Similarly, cohorts 2 and 3 with an association of low neutrophil infiltration and good prognosis were combined and named group 2,3. In the stromal compartment, a trend toward favorable survival was observed in samples with low neutrophil infiltration (*p* = 0.073). In the epithelial compartment, a clear association was observed (*p* = 0.002). Here, the ten-year overall survival rate is only 20% with heavy infiltration and 50% with low neutrophil infiltration of the epithelium. Survival in the two groups of patients with high neutrophil counts was nearly identical for at least 6 years of follow-up (Figure 3G,H). In contrast, the survival of patients with low neutrophil counts was completely different in the two different groups, with the 2.3 group with an extremely low infiltration rate of 3.3 neutrophils/mm^2^ having a very good prognosis. However, the 1,4,5 group with 100 neutrophils/mm^2^ had the worst prognosis (Figure 3G,H). Independence was verified with the Cox regression analysis. The analysis was separately conducted for the two study subgroups. In subgroup 1,4,5, the T category (*p* = 0.011), p16 status (*p* = 0.013) and epithelial neutrophil density (*p* = 0.002) were independent risk factors (Table 3). In subgroup 2,3, only stromal (*p* = 0.027) and intraepithelial neutrophil densities (*p* = 0.050) were independent prognostic factors for overall survival (Table 4).

### 3.5. Characterization of the Two Previously Defined Subgroups

**Figure 4:**
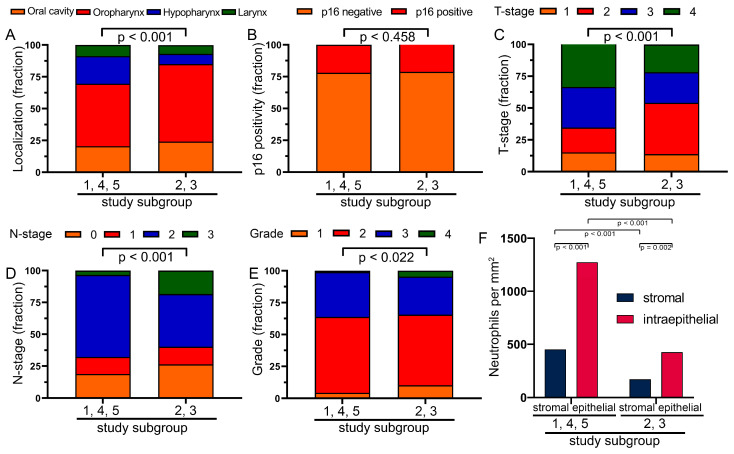
Frequencies of the different characteristics of the two different groups. (**A**) The frequency of different tumor sites in the study subgroups. (**B**) Frequency of p16 status in the study subgroups. (**C**) Proportion of different T stages in both study subgroups. (**D**) Proportion of different N stages in both study subgroups. (**E**) Proportion of different grades in both study subgroups. (**F**) Neutrophil densities in the stromal and epithelial comportment of both study subgroups.

Because of these differences and the contrasting significance of the frequencies in the two subgroups, we were interested in what the differences were between the two groups. Significantly different was the localization with more hypopharynx in group 1,4,5 and more oropharynx in group 2,3 (*p* < 0.001) (Figure 4A). There were only minor differences in HPV positivity (*p* = 0.458) (Figure 4B). The T stage was more advanced in the 1,4,5 subgroup (*p* < 0.001) and the N stage in the 2,3 subgroup (*p* < 0.001) (Figure 4C,D). There were minor differences in grading (*p* = 0.022) (Figure 4E). The most significant difference, however, was in the number of tumor-infiltrating neutrophils. There were 2.6 times more neutrophils in the stromal compartment and 3.0 times more neutrophils in the epithelial compartment in the 1,4,5 subgroup than in the 2,3 subgroup (*p* < 0.001) (Figure 4F). However, in the Kaplan–Meyer analysis, it did not matter whether individual patients had more neutrophils in the stromal compartment than in the epithelial compartment or vice versa (*p* > 0.438). This also did not matter for the two subgroups (*p* = 0.588; *p* = 0.578) or for patients with p16- (*p* = 0.570) or p16+ (*p* = 0.690) tumors.

**Figure 5:**
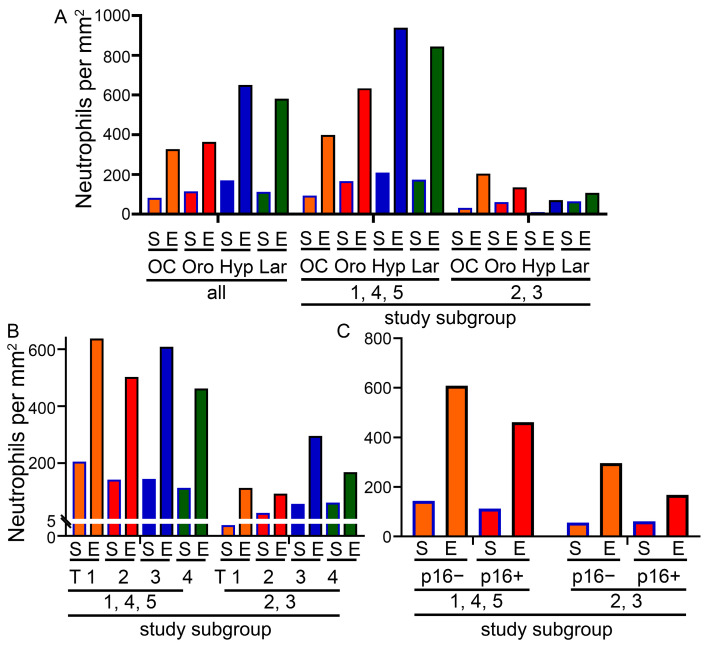
(**A**) Neutrophil density depending on tumor localization and study subgroups. (**B**) Neutrophil density depending on T stage and study subgroups. (**C**) Neutrophil density depending on p16 status and study subgroups. S = stromal, E = epithelial, OC = oral cavity, Oro = oropharynx, Hyp = hypopharynx, Lar = larynx.

Since the neutrophil frequency was significantly different in the two subgroups, we analyzed the frequency according to location, T stage and p16 status. In each of the four locations, there were significantly more neutrophils in study subgroup 1,4,5 compared to subgroup 2,3, in both the stromal and epithelial compartments (Figure 5A). The same is true for the T stage (Figure 5B) and p16 status (Figure 5C). Independent of the location, T stage or p16 status, neutrophilic granulocytes were more common in the epithelial than in the stromal compartment.

**Figure 6:**
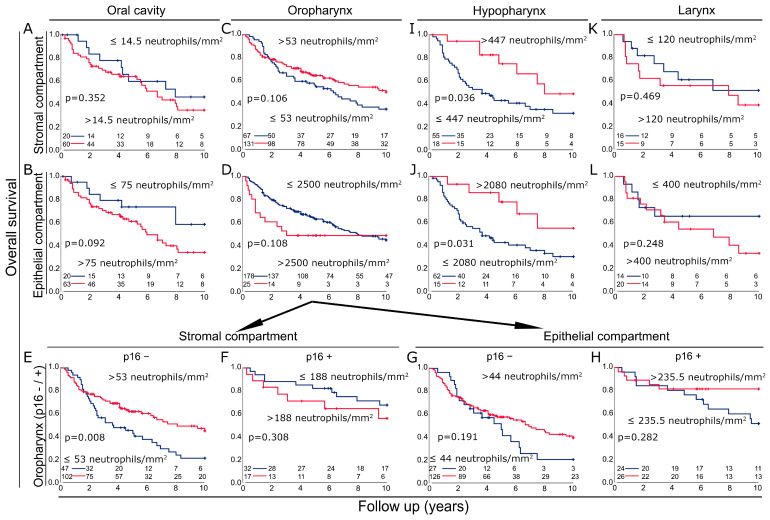
(**A**,**B**) Kaplan–Meier plots of overall survival according to neutrophil densities in stroma and epithelium of tumors of the oral cavity. (**C**,**D**) Kaplan–Meier plots of overall survival according to neutrophil densities in stroma and epithelium of tumors of the oropharynx. (**E**,**F**) Kaplan–Meier plots of overall survival according to neutrophil densities in the stroma of tumors of the oropharynx depending on p16 status. (**G**,**H**) Kaplan–Meier plots of overall survival according to neutrophil densities in the epithelium of tumors of the oropharynx depending on p16 status. (**I**,**J**) Kaplan–Meier plots of overall survival according to neutrophil densities in stroma and epithelium of tumors of the hypopharynx. (**K**,**L**) Kaplan–Meier plots of overall survival according to neutrophil densities in stroma and epithelium of tumors of the larynx.

Because the frequency of neutrophils in the two subgroups differed significantly by site, we investigated the prognostic significance of neutrophils in the four different sites. In the oral cavity, low neutrophil counts were slightly associated with a favorable prognosis in the stromal (*p* = 0.352) and epithelial (*p* = 0.092) compartments (Figure 6A,B). In oropharyngeal cancer, high neutrophil counts were associated with a favorable prognosis in the stromal compartment (*p* = 0.106) and an unfavorable prognosis in the epithelial compartment (*p* = 0.108) (Figure 6C,D). In oropharyngeal cancer, we performed an additional analysis based on p16 status. High neutrophil counts were clearly prognostic in p16-negative HNSCC in the stromal compartment (*p* = 0.008), and in the epithelial compartment, there was a similar trend toward the same effect (*p* = 0.191) (Figure 6E,G). p16 positivity was not associated with a clear prognostic significance of neutrophilic granulocytes in either the stromal or epithelial compartment (*p* > 0.282) (Figure 6F,H). The most striking effect was seen in hypopharyngeal cancer with a clear prognostic benefit of high neutrophil counts in both compartments (*p* < 0.036) (Figure 6I,J). In laryngeal cancer, there was no prognostic significance in either of the two compartments (Figure 6K,L).

**Figure 7:**
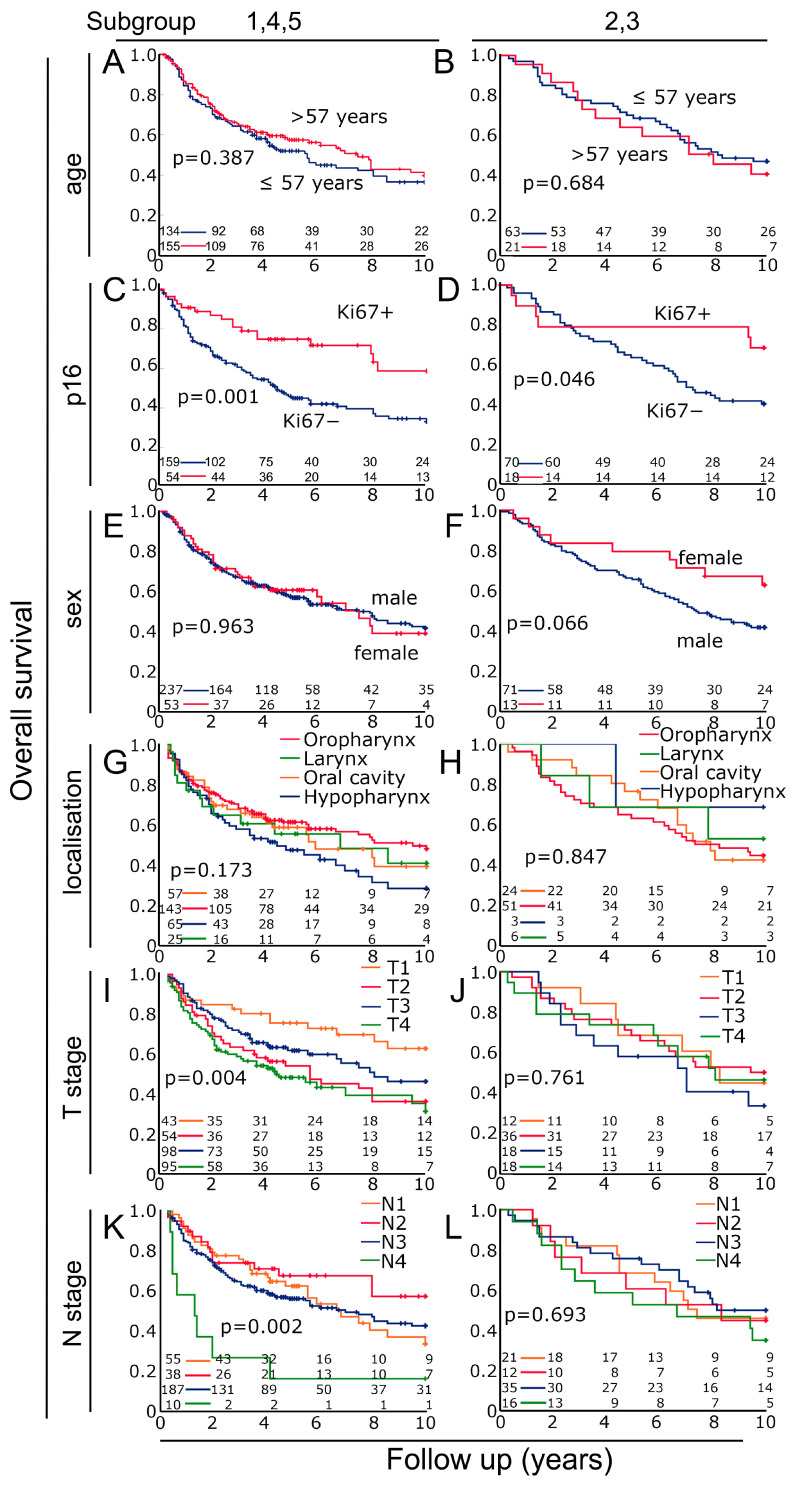
Overall survival of patients with HNSCC in the two cohorts studied separately for different clinical characteristics using Kaplan–Meier plots. Age separated at 57 years (**A**) in groups 1,4,5 and (**B**) in group 2,3. p16 status (**C**) in group 1,4,5 and (**D**) in group 2,3. Males and females (**E**) in group 1,4,5 and (**F**) in group 2,3. Localization sites of HNSCC (**G**) in group 1,4,5 and (**H**) in group 2,3. Different T stages (**I**) in group 1,4,5 and (**J**) in group 2,3. Different N stages (**K**) in group 1,4,5 and (**L**) in group 2,3.

Finally, for the two subgroups, the classical factors reported to impact prognosis were compared. In subgroup 1,4,5, p16 status (*p* = 0.001), T stage (*p* = 0.004) and N stage (*p* = 0.002) were highly prognostic. In contrast, in subgroup 2,3, only p16 status (*p* = 0.046) and sex (*p* = 0.066) were prognostically borderline (Figure 7). In comparison, these factors for the cohort as a whole are shown in Supplementary Figure S5. Additionally, the numbers of neutrophils in the different locations and subgroups were analyzed (Supplementary Table S1). There were no clear effects in the different groups. Older patients (>57 years) had more neutrophils than younger patients. In the more advanced cancers, however, there was no clear increase in neutrophils.

## 4. Discussion

Data on the role of neutrophils in the different sites of HNSCC are still relatively scarce [12]. Surprisingly, in our study, we observed that high densities of tumor-infiltrating neutrophils were associated with favorable survival in some cohorts and unfavorable survival in others. This was true for both the stromal and epithelial compartments of the tumor. Both compartments were analyzed completely separately and this proves again that this dichotomy is really true. This opposite effect may be related to the fact that neutrophils, like macrophages, polarize into two different states [26]: a cytotoxic anti-tumor N1 state and an immunosuppressive pro-tumor N2 state [27]. Unfortunately, no suitable markers exist so far to identify these characteristics of neutrophils and it is not possible to identify the specific function of these inflammatory cells [13]. However, considering that, in our study, in three cohorts high numbers of neutrophils were associated with a favorable prognosis, it could be assumed that in these cohorts neutrophils were more likely to exert a cytotoxic function. Moreover, in the study cohort 2,3, high numbers of neutrophils were associated with an unfavorable prognosis, indicating a more immunosuppressive phenotype. Compared to cohort 2,3, there were significantly more neutrophils in cohort 1,4,5. This could be interpreted as inflammation in cohort 1,4,5 and it could be possible that in inflamed cancers, the N1 polarization state, and therefore an anti-tumor effect, is more common. However, it may also be related to other inflammatory cells that act in an anti-tumorigenic manner. Conversely, the non-inflamed 2,3 cohort, in which fewer neutrophils were present, could have a higher N2 fraction. This may have a stronger immunosuppressive effect on the tumor microenvironment and may lead to a worse prognosis for patients.

For other inflammatory cells, such as memory T cells (CD45R0+) [28] and cytotoxic (CD8+) and regulatory T cells (FoxP3+) [9], higher infiltration of inflammatory cells was present in the stromal tumor compartment than in the epithelial tumor compartment, respectively, in the identical cohort. Nine different tumor-infiltrating inflammatory cell types were examined both before and after radiotherapy in cohort 3. Only dendritic CD1a+ cells had a higher cell density in the intraepithelial compartment than in the stromal compartment. All other types of inflammatory cells were more numerous in the stromal compartment than in the epithelial compartment. This was true for CD8+ T lymphocytes, CD3+ T lymphocytes, CD4+ T helper cells, regulatory T cells, B cells, macrophages and Granzyme B+ cells [29]. In rectal cancer, we measured predominantly stromal CD8+ T cells and regulatory T cells, which were very close to the epithelial–stromal interface, possibly indicating that these cells were not able to invade the epithelial compartment. This proximity to the epithelial compartment was prognostically relevant [30]. Here, the reverse is true. Neutrophil granulocytes had much higher infiltration rates in the tumor epithelium compared to the stromal compartment. In the other cell types, it appears that the epithelial compartment may actively prevent cell immigration. In group 2,3, with low infiltration rates and the best overall survival, there were 16× fewer neutrophils in the epithelium than in the stromal compartment.

Here, only very few neutrophils were found in the epithelial compartment, namely 3.3 neutrophils per mm^2^, which was associated with favorable survival. In both the 2,3 and 1,4,5 groups with high neutrophil infiltration of 600 to 1500 cells/mm^2^, survival was intermediate. An infiltration rate of at least 100 cells/mm^2^ in the epithelial compartment, which leads to the worst prognosis in the low neutrophil group 1,4,5, suggests that these are mainly immunosuppressive cells. Our results suggest that neutrophils may be one of the few types of inflammatory cells that are able to infiltrate the tumor epithelium relatively easily, while tumors often exclude other inflammatory cells from the epithelial compartment.

It is unclear why we observed such an impressive difference in terms of prognostic significance of neutrophils between the two groups 1,4,5 and 2,3. There was no clear correlation with clinical features or treatment modality. The main difference was that in the subgroup in which low neutrophil counts were favorable, overall neutrophil counts were significantly lower and median overall survival was longer. In addition, the proportion of hypopharyngeal cancers, which had the worst prognosis, was lower in this cohort. However, this does not explain the differences we observed between the two cohorts.

In a recent review by Wondergem et al., it was shown that the meaning of tumor-infiltrating neutrophils in HNSCC is still widely unclear [12]. Our results have clearly demonstrated that in hypopharyngeal cancer and p16-negative oropharyngeal cancer, a high percentage of neutrophil granulocytes was associated with a favorable prognosis. This is true for all our cohorts. In contrast, neutrophil counts were not prognostically meaningful in all other locations of HNSCC. Thus, in hypopharyngeal cancer and p16-negative oropharyngeal cancer, the association between high neutrophil counts and favorable prognosis appears to be a general effect independent of other factors. Conversely, two studies found a worse prognosis associated with high neutrophil levels in oropharyngeal and hypopharyngeal cancer [23,24]. CD66b+ neutrophils were studied in patients with advanced oropharyngeal or hypopharyngeal cancer with predominantly definitive radiochemotherapy. Here, T4 cancers had the highest tumor infiltration scores. Patients with intermediate or high infiltration scores had worse survival compared to patients with no or low infiltration scores [24]. Another study in oropharyngeal or hypopharyngeal cancer compared low versus high infiltration of CD66b+ neutrophils. High neutrophil infiltration tended to be associated with an unfavorable prognosis. In cancers with N2c/N3 local metastasis, high numbers of neutrophils were counted [23].

One aspect of neutrophil significance that has not been considered here so far and should be further investigated is neutrophil extracellular traps (NETs). NETs are web-like structures composed of DNA, histones and proteins [31]. They are released from activated neutrophils and are implicated in tumor initiation and progression in cancer [32]. NETs should be studied to have a better overall picture of neutrophil function.

### Limitations

A major strength of our study was the large cohort of 397 patients and the long-term survival data of 10 years for the majority of cases. In addition, we achieved a high degree of precision by using a semiautomated counting method separately for the stromal and intraepithelial compartments. Weaknesses were the retrospective design, heterogenic gender distribution, tumor location and treatment modalities, which could have had an impact on the relationship between neutrophil infiltration and survival. Additionally, we only measured cell densities in one to three TMA spots per patient with an area of 3.1 to 9.3 mm^2^, which might not fully account for the possibility of uneven cell density distribution inside the tumor. However, a very high concordance between TMA spots and whole tissue sections in immunohistochemical studies has been reported. Furthermore, clinical characteristics were not available for all patients.

## 5. Conclusions

It is possible that tumor-infiltrating neutrophil granulocytes in HNSCC may contribute to a favorable or unfavorable prognosis. Cancers with an exclusion of neutrophils from the epithelial compartment were associated with prolonged survival. In oropharyngeal and hypopharyngeal HPV-negative cancer, high neutrophil infiltration rates were clearly associated with a favorable prognosis.

## Figures and Tables

**Table 1:** Clinical characteristics of 397 HNSCC patients.

	Variable	Cohort *n* (%)
Sex:	Male	330 (83%)
	Female	67 (17%)
Age:	Median (range)	57 (27–81)
Primary tumour:	T Stage 1	59 (14.9%)
	2	96 (24.2%)
	3	119 (30.0%)
	4	123 (31.0%)
Lymph node status:	N Stage 0	76 (19.1%)
	1	52 (13.1%)
	2	240 (60.5%)
	3	26 (6.5%)
Metastasis:	M Stage 0	392 (98.7%)
	1	5 (1.3%)
Grade:	Grading 1	22 (5.5%)
	2	231 (58.2%)
	3	136 (34.3%)
	4	8 (2%)
Stage:	UICC Stage I	12 (3.0%)
	II	26 (6.5%)
	III	47 (11.8%)
	IV	293 (73.8%)
	unknown	19 (4.8%)
Location:	Oral Cavity	83 (21%)
	Oropharynx	203 (51%)
	Oropharynx p16 pos.	50 (13%)
	Oropharynx p16 neg.	153 (38%)
	Hypopharynx	77 (19%)
	Larynx	34 (9%)
Treatment:	definitive	47 (11.8%)
	adjuvant	325 (81.9%)
	neoadjuvant	25 (6.3%)
Tumor:	p16−	232 (78.1%)
	p16+	65 (21.9%)

UICC = Union for International Cancer Control, R(C)T = radio or radiochemotherapy, adjuvant = surgery followed by R(C)T, neoadjuvant = R(C)T followed by surgery, definitive = exclusive R(C)T.

**Table 2:** This study included six HNSCC trials in which tissue samples were obtained from patients diagnosed between 1984 and 2015. Adjuvant = surgery followed by R(C)T, neoadjuvant = R(C)T followed by surgery, definitive = only R(C)T.

Study Cohort	Study From–Until	Radiation Therapy	n (397)	Median Follow-Up (Years)
0	1998–2003	adjuvant	19	5.6
1	1998–2005	definitive	47	3.4
2	1984–1998	adjuvant	59	7.4
3	1997–2004	neoadjuvant	25	7.9
4	2002–2010	adjuvant	137	4.5
5	2010–2015	adjuvant	110	3.9
all	1998–2015	---	397	4.3

**Table 3:** Univariate and multivariate analysis of overall survival according to Cox’s proportional hazards model. HNSCC carcinoma subgroup 1,4,5.

	Univariate Analysis			Multivariate Analysis		
Variable	Hazard Ratio	95% C.I.	*p*	Hazard Ratio	95% C.I.	*p*
Age, years (younger 57 years [n = 80] vs. older 57 years [n = 73])	1.255	0.799–1.973	0.324	---	---	---
Sex (male [n = 125] vs. female [n = 28])	0.763	0.438–1.33	0.341	---	---	---
p16 (negative [n = 118] vs. positive [n = 35])	2.093	1.13–3.876	**0.019**	2.133	1.169–3.89	**0.013**
T category (T1/T2 [n = 100] vs. T3/T4 [n = 53])	0.535	0.329–0.868	**0.011**	0.557	0.354–0.875	**0.011**
N category (N0 [n = 20] vs. N+ [n = 133])	1.107	0.596–2.056	0.748	---	---	---
Grading (1 + 2 [n = 64] vs. 3 + 4 [n = 89])	0.763	0.471–1.235	0.270	0.800	0.505–1.265	0.339
Neutrophils epithelial (low [n = 63] vs. high [n = 90])	1.528	0.855–2.731	0.152	2.011	1.29–3.135	**0.002**
Neutrophils stomal (low [n = 72] vs. high [n = 81])	1.415	0.783–2.558	0.250	1.713	0.978–3.001	0.060

**Table 4:** Univariate and multivariate analysis of overall survival according to Cox’s proportional hazards model. HNSCC carcinoma subgroup 2,3.

	Univariate Analysis			Multivariate Analysis		
Variable	Hazard Ratio	95% C.I.	*p*	Hazard Ratio	95% C.I.	*p*
Age, years (younger 57 years [n = 55] vs. older 57 years [n = 18])	1.054	0.498–2.23	0.891	---	---	---
Sex (male [n = 61] vs. female [n = 12])	2.608	0.737–9.232	0.137	2.929	0.894–9.595	0.076
p16 (negative [n = 58] vs. positive [n = 15])	1.423	0.475–4.262	0.529	1.339	0.472–3.793	0.583
T category (T1/T2 [n = 59] vs. T3/T4 [n = 14])	1.285	0.548–3.014	0.564	0.819	0.362–1.854	0.632
N category (N0 [n = 17] vs. N+ [n = 56])	0.857	0.399–1.842	0.693	---	---	---
Grading (1 + 2 [n = 49] vs. 3 + 4 [n = 24])	0.959	0.481–1.91	0.905	---	---	---
Neutrophils epithelial (low [n = 46] vs. high [n = 27])	0.532	0.282–1.003	0.051	0.500	0.27–0.926	**0.027**
Neutrophils stomal (low [n = 33] vs. high [n = 40])	0.537	0.27–1.069	0.077	0.530	0.281–1	**0.050**

## Data Availability

The data presented in this study are available upon request from the corresponding author.

## Supplementary Materials

The following supporting information can be downloaded at: https://www.mdpi.com/article/10.3390/biom14020205/s1, Figure S1: (A) Isotype controls were performed with biotinylated goat anti-mouse IgM secondary antibodies, omitting the primary antibodies. Otherwise, the staining procedure was identical to the primary antibody staining. (B) The staining procedure was performed in accordance with the protocol used in the study. Control studies have been performed on tissue from the jejunum. The scale bar in (B) indicates 100 μm. Figure S2: Kaplan–Meier plots of overall survival in the HNSCC cohort according to neutrophil densities depending on (A) age, (B) p16 status, (C) sex, (D) tumor localization, (E) T stage and (F) N stage. Figure S3: HNSCC tissue stained for CD66b+ neutrophils. (A) The whole spot of the tissue microarray with the blue square marking the area of the enlarged section, (B) a section with separately labeled epithelial and stromal compartments. (red), (C) the section without labeled compartments. A tissue with neutrophils predominantly in the stromal compartment and to a much lesser extent in the epithelial compartment. The scale bar in (A) indicates 500 μm and in (C) 50 μm. Figure S4: HNSCC tissue stained for CD66b+ neutrophils. (A,D,G) The whole spots of the tissue microarray with the blue square marking the area of the enlarged sections, scale bars indicate 500 μm. (B,E,H) sections with separately labeled epithelial and stromal compartments (red). (C,F,I) sections without labeled compartments. (A–C) A tissue with neutrophils predominantly in the epithelial compartment. (D–F) A tissue with neutrophils predominantly in the epithelial compartment. (G–I) A tissue with neutrophils in the stromal and epithelial compartments. The scale bars indicate 50 μm. Figure S5: Kaplan–Meier plots of overall survival in the HNSCC cohort according to neutrophil densities depending on the stromal to epithelial ratio in (A) all patients, (B) the 1,4,5 cohort, (C) the 2,3 cohort, (D) p16 negative patients and (E) p16 positive patients. Table S1: Mean neutrophils in the stromal and epithelial compartments of different locations and different subgroups. Number of patients in brackets.
