# Supplementary material for: Neutrophils in HNSCC Can Be Associated with Both a Worse or Favorable Prognosis
Source: Biomolecules. 2024 Feb 9;14(2):205. doi: 10.3390/biom14020205 (PMC10886850; doi:10.3390/biom14020205)
Supplement: Supplementary file 1 [file biomolecules-14-00205-s001.zip › biomolecules-2814269-supplementary.pdf]

Neutrophils in HNSCC can be associated with both a worse or favorable prognosis

Hendrik Brunkhorst [1,2,†], Sören Schnellhardt [1,2,3,†], Maike Büttner-Herold [4], Christoph Daniel [4], Rainer Fietkau [1,2] and Luitpold Distel [1,2,*]

1 Department of Radiation Oncology, Universitätsklinikum Erlangen, Friedrich-Alexander-Universität Erlangen-Nürnberg, 91054 Erlangen, Germany
2 Comprehensive Cancer Center Erlangen-EMN (CCC ER-EMN), 91054 Erlangen, Germany
3 Department of Radiotherapy and Radiation Oncology, Saarland University Medical Center, 66421 Homburg, Germany
4 Department of Nephropathology, Institute of Pathology, Universitätsklinikum Erlangen, Friedrich-Alexander-Universität Erlangen-Nürnberg, 91054 Erlangen, Germany
* Correspondence: luitpold.distel@uk-erlangen.de; Tel.: +49-9131-853-2312; Fax: +49-9131-853-9335
† These authors contributed equally to this work.

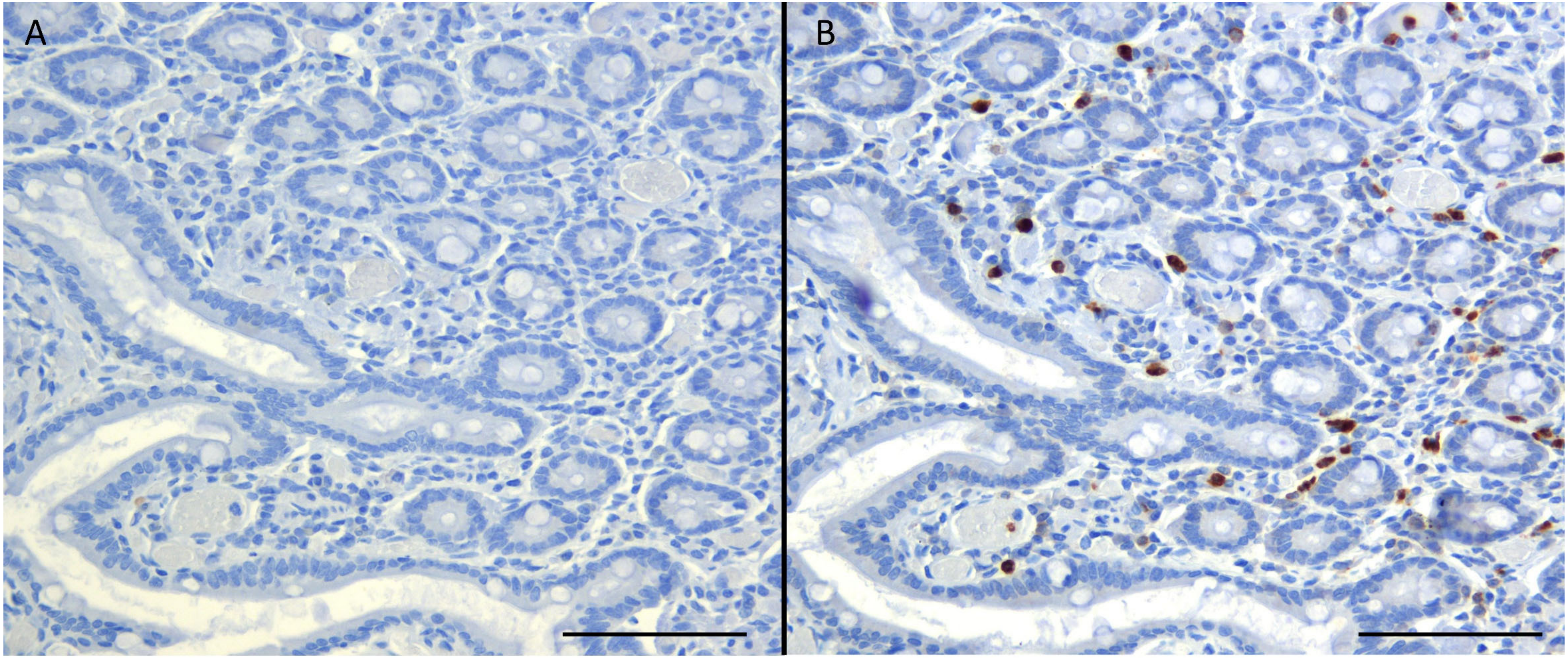

Supplementary Figure S1: (A) Isotype controls were performed with biotinylated goat anti-mouse IgM secondary antibodies, omitting the primary antibodies. Otherwise, the staining procedure was identical to the primary antibody staining. (B) The staining procedure was performed in accordance with the protocol used in the study. Control studies have been performed on tissue from the jejunum. The scale bar in (B) indicates 100 µm.

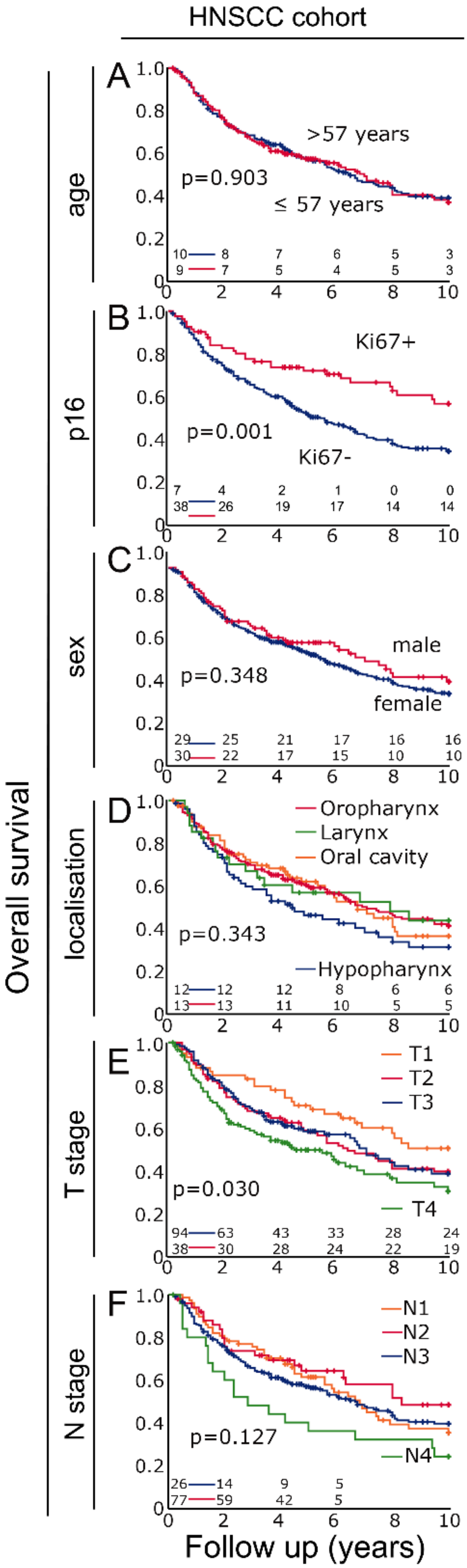

Supplementary figure S2 Kaplan-Meier plots of overall survival in the HNSCC cohort according to neutrophil densities depending on (A) age, (B) p16-status, (C) sex, (D) tumor localization, (E) T-stage and (F) N-stage.

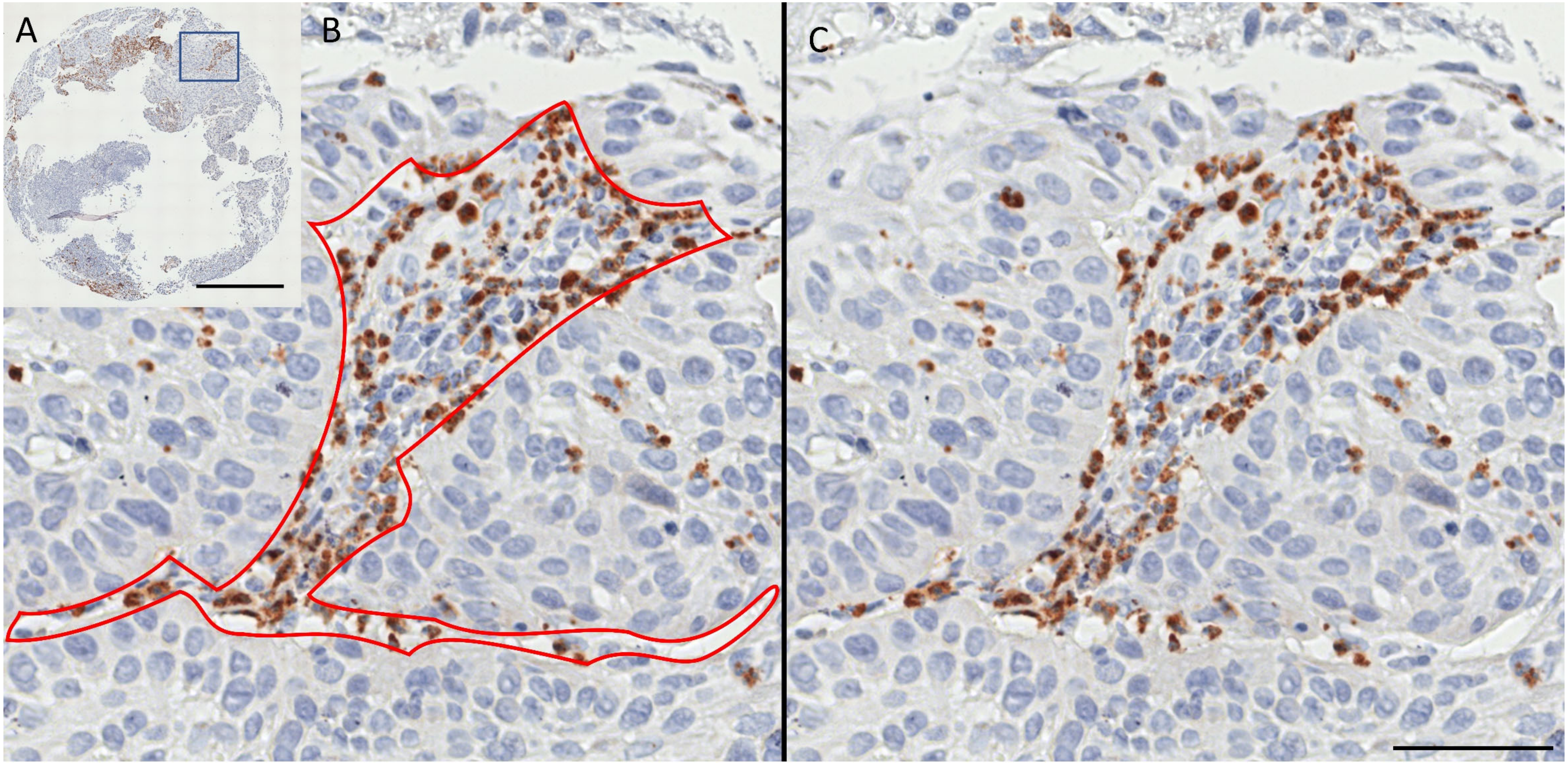

Supplementary Figure S3: HNSCC tissue stained for CD66b+ neutrophils. (A) The whole spot of the tissue microarray with the blue square marking the area of the enlarged section, (B) a section with separately labeled epithelial and stromal compartments. (red), (C) the section without labeled compartments. A tissue with neutrophils predominantly in the stromal compartment and to a much lesser extent in the epithelial compartment. The scale bar in (A) indicates 500 µm and in (C) 50 µm.

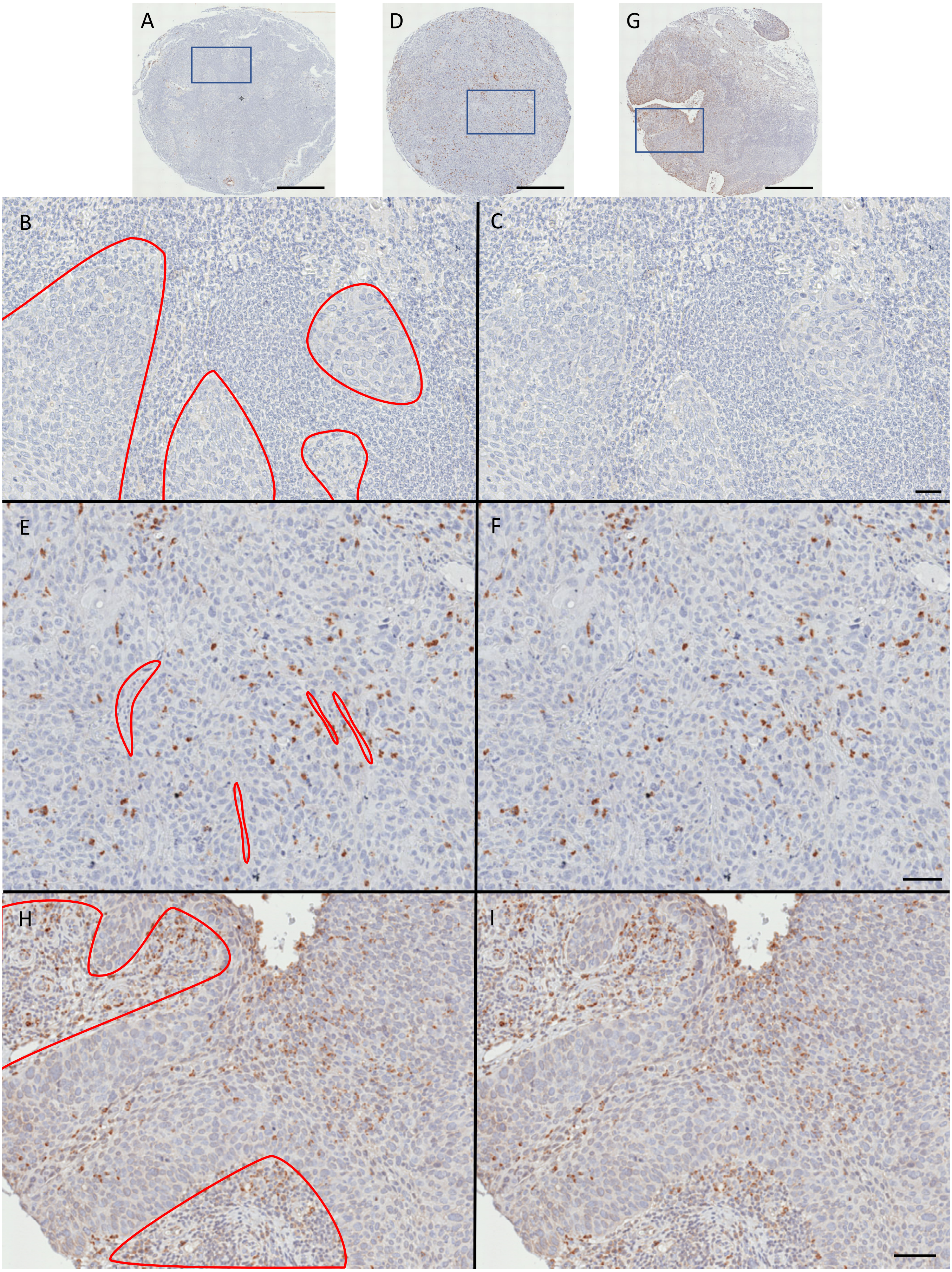

Supplementary Figure S4: HNSCC tissue stained for CD66b+ neutrophils. (A, D, G) The whole spots of the tissue microarray with the blue square marking the area of the enlarged sections, scale bars indicates 500 µm. (B, E, H) sections with separately labeled epithelial and stromal compartments (red). (C, F, I) the section without labeled compartments. (A, B, C) A tissue with neutrophils predominantly in the epithelial compartment. (D, E, F) A tissue with neutrophils predominantly in the epithelial compartment. (G, H, I) A tissue with neutrophils in the stromal and epithelial compartments. The scale bars indicate 50 µm.

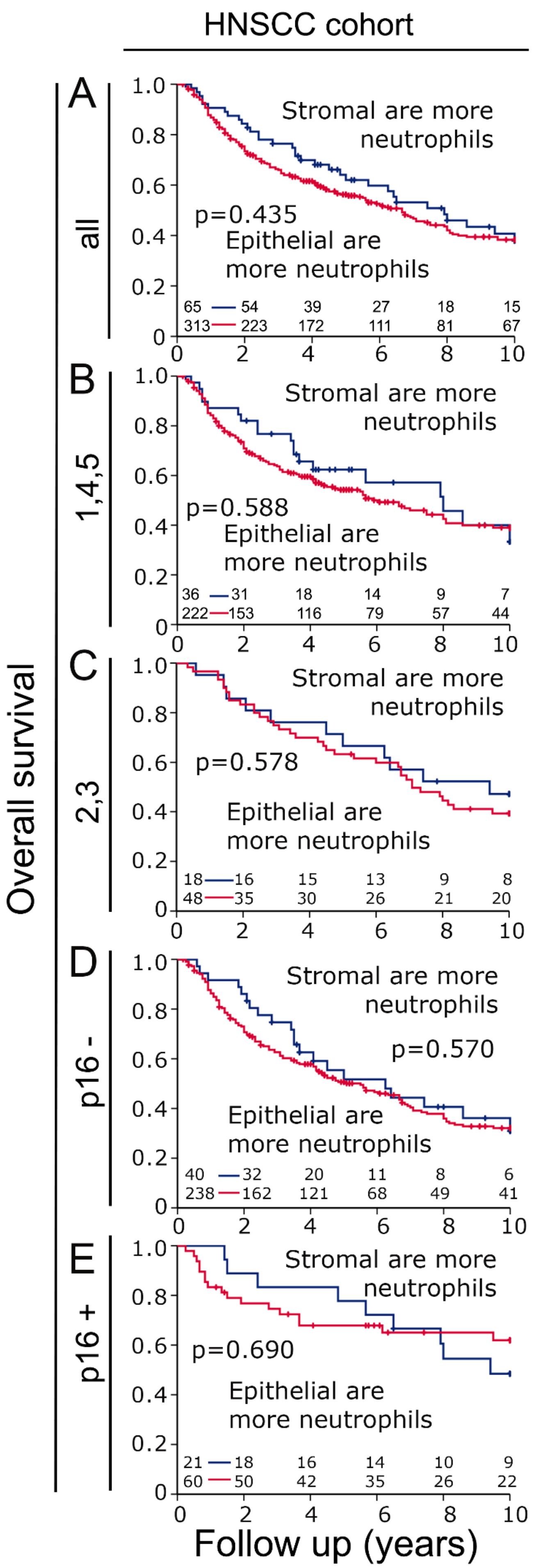

Supplementary figure S5 Kaplan-Meier plots of overall survival in the HNSCC cohort according to neutrophil densities depending on the stromal to epithelial ratio in (A) all patients, (B) the 1,4,5 cohort, (C) the 2,3 cohort, (D) p16 negative patients and (E) p16 positive patients.

Supplementary table S1: Mean neutrophils in the stromal and epithelial compartments of different locations and different subgroups. Number of patients in brackets.

		all locations		oral cavity		oropharynx		hypopharynx		larynx	
	variable	stromal	epithelial	stromal	epithelial	stromal	epithelial	stromal	epithelial	stromal	epithelial
	all	813.7 (387)	1478.8 (397)	394.5 (80)	1648.3 (83)	823.6 (198)	1295.4 (203)	1161.4 (73)	1794.1 (77)	559.3 (31)	1252.4 (34)
age	≤ 57	512.2 (205)	1291.9 (214)	310.1 (46)	1436.3 (48)	593.9 (101)	1234.4 (103)	540 (40)	1265.3 (43)	356.4 (18)	1344.7 (20)
	>57	1059.8 (176)	1647.5 (182)	482.9 (34)	1922.7 (35)	1004.9 (96)	1321.7 (99)	1606.9 (33)	2148.3 (34)	771.1 (13)	1130 (14)
sex	male	861.2 (323)	1466.6 (330)	390.8 (63)	1739.3 (65)	894.6 (164)	1358.8 (169)	1193 (62)	1534.8 (64)	577.9 (29)	1291.4 (32)
	female	506.4 (64)	1543.9 (67)	420.3 (17)	1298.9 (18)	211.9 (34)	897.5 (34)	1001.5 (11)	2819.4 (13)	4.9 (2)	238.2 (2)
T stage	T1	1232.3 (58)	1873.3 (59)	603.8 (9)	3302.1 (10)	414.8 (39)	1214 (39)	2940.1 (9)	1732.3 (9)	0 (1)	0 (1)
	T2	412.8 (94)	1477.8 (96)	198.1 (23)	673.8 (23)	393.2 (52)	1009.3 (53)	648 (14)	2960.7 (14)	218 (5)	467.3 (6)
	T3	877.9 (115)	1333.7 (119)	445.5 (18)	1355.3 (18)	1059.3 (60)	1455.6 (62)	697.1 (24)	1162.1 (25)	733.5 (13)	1094 (14)
	T4	745.8 (115)	1390.4 (123)	384 (30)	1306.9 (32)	1047.6 (47)	1382.9 (49)	426.4 (26)	1425.9 (29)	484.3 (12)	1649.9 (13)
N stage	N0	811.8 (75)	1637.2 (79)	464.6 (16)	2800.6 (17)	994.5 (38)	788.2 (40)	722.6 (17)	1573.9 (17)	162 (4)	265.8 (5)
	N1	371 (51)	909.7 (52)	153.2 (13)	682.4 (13)	413.6 (30)	909.9 (30)	512.4 (6)	794 (6)	222.3 (2)	1567.1 (3)
	N2	904.9 (230)	1542.9 (240)	417.7 (50)	1209.3 (52)	859.6 (111)	1481.2 (114)	1396 (45)	2002.1 (49)	624.8 (24)	1300.8 (25)
Grade	Grade 1	1185.2 (21)	961.6 (22)	76.3 (8)	331.6 (8)	1690.5 (10)	1204.2 (11)	23 (2)	14.6 (2)	0 (1)	0 (1)
	Grade 2	903.6 (222)	1533.8 (231)	451.3 (48)	1820.1 (51)	781.2 (113)	1132.7 (113)	1539.9 (39)	2044.2 (43)	646.1 (22)	1420.5 (24)
	Grade 3	546.8 (131)	1426.6 (136)	317.3 (24)	1484.8 (24)	669.6 (71)	1456.3 (75)	399 (28)	1505.1 (28)	187.5 (8)	568 (9)
	Grade 4	1003.9 (8)	1903 (8)	- (0)	- (0)	1384.7 (4)	2765.1 (4)	123.6 (4)	780.4 (4)	- (0)	- (0)
